# Prevalence of and risk factors for *Helicobacter pylori* infection in children under 64 months in Thimphu, Bhutan, and introducing the new in-house immunochromatography test kit: a cross-sectional study

**DOI:** 10.1186/s13099-025-00715-2

**Published:** 2025-06-04

**Authors:** Passang Lhamo Sherpa, Takashi Matsumoto, Kinley Tshering, Birendra Pradhan, Junko Akada, Yoshio Yamaoka

**Affiliations:** 1 Department of Environment and Preventive Medicine, Faculty of Medicine, Oita University, Yufu City, 879-5598 Japan; 2 Lungtenphu Hospital, Thimphu, Bhutan; 3 Department of Pathology and Laboratory, Jigme Dorji Wangchuck National Referral Hospital, Thimphu, Bhutan; 4 Department of Medicine, Gastroenterology and Hepatology Section, Baylor College of Medicine, Houston, TX 77030 USA

**Keywords:** *Helicobacter pylori*, Prevalence and risk factors, Children, Stool antigen test, Immunochromatography, Bhutan

## Abstract

**Background:**

*Helicobacter pylori* (*H. pylori)* is a lifelong infection, often acquired in childhood and persisting throughout life, that can lead to serious gastric diseases, including gastric cancer in adults. While asymptomatic in most children, it may cause extraintestinal manifestations affecting growth, necessitating distinct pediatric management strategies—particularly in countries with a high risk of gastric cancer. Accurate diagnosis is critical in high-risk populations. The stool antigen test is a reliable, non-invasive method for young children. Despite Bhutan’s high *H. pylori* burden, diagnostic tools remain scarce. This study aimed to determine the prevalence and risk factors of *H. pylori* infection in Bhutanese children and validate a new in-house immunochromatography test (the A-ICT) kits.

**Methods:**

A cross-sectional study was conducted in 2023 among children under 64 months of age at three immunization clinics in Thimphu. *H. pylori* antigen in stool was detected using an ICT kit. After obtaining informed consent, parents completed questionnaires. Data were analyzed using STATA version 14.2 and R version 4.4.1.

**Results:**

A total of 226 children (mean age 33.28 months) participated in the study. The A-ICT kit showed high concordance with the commercial kit (Kappa 0.84 [95% CI: 0.78–0.89]) and excellent sensitivity (0.96) and specificity (0.95). The prevalence of *H. pylori* was 19.54% (95% CI:14.95–24.83). Risk factors included increasing age, having two or more siblings, and fathers who were farmers/wagers, and who worked in government/private sector. Children who were fed with or who ate using a spoon had a significantly lower risk of *H. pylori* infection than those who were fed or ate with fingers *(p* < 0.05).

**Conclusions:**

The A-ICT kit demonstrated remarkable sensitivity and specificity. Improvements in hygiene and sanitation related to child feeding practices are essential. Awareness programs should target large families and individuals employed in the formal sector, including both household and workplace settings. The validation of the A-ICT is a significant step toward a gastric cancer prevention program that facilitates early diagnosis and *H. pylori* eradication. The test kit is highly recommended for *H. pylori* screening and the confirmation of eradication post-treatment given its accuracy, rapidity, and simplicity in execution.

**Supplementary Information:**

The online version contains supplementary material available at 10.1186/s13099-025-00715-2.

## Background

*Helicobacter pylori* (*H. pylori*) is a globally prevalent bacterial infection that often begins in early childhood and, if untreated, persists throughout life. *H. pylori* infection is the leading cause of chronic gastritis and is critical in the pathogenesis of peptic ulcer disease, gastric adenocarcinoma, and mucosa-associated lymphoid tissue lymphoma [1, 2]. Although gastric diseases primarily manifest in adulthood, *H. pylori* infection during childhood has been associated with extraintestinal complications including iron deficiency anemia, idiopathic thrombocytopenic purpura, and growth retardation [3]. Given the potential long-term health consequences, early detection and prevention are crucial, especially in high-risk populations with elevated GC incidence.

The primary mode of *H. pylori* transmission remains under investigation, but person-to-person spread primarily through oral-oral or fecal-oral pathways is widely accepted as the main route. Household transmission is considered a significant factor; infected parents or siblings are often the source of infection in children [4, 5]. Socioeconomic factors such as overcrowding, poor sanitation, and unhygienic food-handling practices further contribute to the spread of *H. pylori* [6–8].

Reliable and accessible diagnostic tools are essential for effective *H. pylori* management. Although invasive methods such as endoscopic biopsy and histology are the gold standards for diagnosis, these methods are impractical for large-scale screening, particularly in pediatric populations. Non-invasive diagnostic tests including the urea breath test and stool antigen test (SAT) are preferred in children [9–11]. Among these, the SAT is advantageous for its high sensitivity and specificity, affordability, and ease of execution because it does not require fasting or specialized laboratory infrastructure [12, 13], making it an ideal tool for resource-limited settings. However, access to the SAT remains limited in Bhutan, and alternative, cost-effective diagnostic solutions are required to expand *H. pylori* detection efforts.

Globally, *H. pylori* prevalence in children varies significantly, ranging from 20% to over 50%; higher rates are observed in low- and middle-income countries where sanitation, infrastructure and access to healthcare remain inadequate [14]. In Bhutan, previous studies have reported a high *H. pylori* prevalence, with infection rates among children reaching 66% in earlier surveys [15]. However, improvements in public health measures and sanitation and an increased awareness of hygiene practices may have influenced these rates over time. Additionally, Bhutan’s national GC prevention efforts [16, 17], which have focused on *H. pylori* eradication in adults, may have indirectly contributed to reducing transmission in younger populations. Despite these efforts, accurate epidemiological data on *H. pylori* prevalence in young children remain scarce, underscoring the need for updated studies. Moreover, the age-dependent pattern of infection in children likely reflects recent transmission from household members, particularly mothers [18, 19]. Therefore, the prevalence of infection among young children may serve as a proxy indicator of parental—especially maternal—infection, particularly following eradication programs.

Despite the high prevalence of *H. pylori* infection in Bhutan, diagnostic resources remain limited, particularly for non-invasive testing. Current methods depend on facility levels, with urease tests and UBT available only in selected hospitals and often inconsistently [16, 17]. Introducing the ADTEC in-house immunochromatography stool antigen test (A-ICT) offers a simple, accurate, and non-invasive diagnostic option with high feasibility for decentralized settings, ideal for Bhutan’s resource-limited settings. The A-ICT also offers practical advantages over conventional commercial kits (e.g., O-ICT), including its potential for local production in Bhutan, cost-effectiveness, reduced packaging, and minimal training requirements.

Therefore, this study aims to [1] determine the prevalence of *H. pylori* infection among children under 6 years of age in Thimphu, Bhutan [2], investigate potential risk factors for infection, and [3] evaluate the performance of a newly developed in-house immunochromatography stool antigen test (the A-ICT). If validated, this test could offer a reliable, rapid, and affordable alternative to existing diagnostic tools to improve early detection and intervention strategies in Bhutan. These findings may contribute to shaping national *H. pylori* screening and eradication programs and ultimately preventing GC.

## Methods

### Study site

Bhutan has a three-tiered healthcare system that consists of primary health centers (PHCs) at the basic level, district and general hospitals at the intermediate level, and referral hospitals at the top level [20]. The PHCs are the grassroots care providers in the community and provide preventive care such as child immunization, growth monitoring, and child nutrition. We selected one PHC (Kuzugchen PHC) and two general hospitals (Gidakom Hospital in the industrial area in the southern periphery of Thimphu, and Lungtenphu Hospital, a military hospital in the heart of Thimphu City) within Thimphu Dzongkhag (prefecture). These health facilities are within a radius of 30 km and are in their respective catchment areas.

### Study population, sample size and design

This cross-sectional study assessed prevalence and risk factors for *H. pylori* infection within a single cohort of children; no external control group was used. Comparisons were made between *H. pylori* positive and negative children. The study was conducted in Thimphu during May–June 2023 in three health centers mentioned above. The sample was derived from a population of children those who received immunization services in the three health centers with a mean age of 33.28 (range: 0.30–64.36 ± 18.06) months. To calculate the required sample size, we used the prevalence rate (66%) from a previous serological study conducted in Bhutan [15] with a precision of 6% at alpha level 0.05 using the following formula [21]: $\:N=\:\frac{{Z}^{2}P\:\left(1-P\right)}{{d}^{2}}$. The calculation yielded a sample size of approximately 240 participants. Initially, we randomly selected and registered 290 children across three clinics based on clinic attendance during the recruitment period. The inclusion criteria were healthy children (children who were healthy and visited the clinic solely for routine immunizations and nutritional counseling) who attended the immunization clinic, whereas the exclusion criteria were children who came to the clinic because they were unwell (not for the purpose of immunization) or whose parents refused participation. Risk factors were identified using data from parent-reported questionnaire data, analyzed through logistic regression. First, all of the parents/guardians signed an informed consent form. Next, they completed a questionnaire that collected sociodemographic and environmental information. To detect *H. pylori* infection, we collected stool samples from the children and stored them at − 80^°^C before testing.

### Questionnaire

To identify potential risk factors for *H. pylori* infection in this cross-sectional study, we used a structured questionnaire completed by parents. All of the parents/guardians answered the questionnaire by themselves or with the help of two specifically trained enumerators. The questionnaire was adapted from the previous National Nutrition Survey 2015, Bhutan. Five sections were included in the questionnaire. The first section collected general demographic characteristics of the child (e.g., age, sex, anthropometric measurement, birth order, number of siblings, number of people in the household, child feeding methods, whether the child had any GI-related problems in the past month before the survey and place of recruitment). The second section queried dietary habits (e.g., the frequency of consumption of meat, salt-containing foods, vegetables, and fruits). Section three included socioeconomic variables (e.g., parents’ educational and employment status). The fourth section covered behavioral characteristics (e.g., water source and supply, whether water was boiled before consumption, the incidence of hand washing, and toilet type and location), and the last section queried the parents’ *H. pylori* status (whether they had ever tested for *H. pylori* and test results). Responses were directly entered into SPSS version 25 (IBM Corp., Armonk, NY, USA). The variables generated from the questionnaire were used in logistic regression analysis to compare children who tested positive and negative for *H. pylori.*

### Stool sample

Parents were given a clean, empty tube for stool sample collection and instructions on collecting the child’s stool. Parents were asked to store any samples collected the night before in the refrigerator and bring samples to the clinic the following day. Upon receipt, the samples were placed in an ice box and transported to the Royal Center for Disease Control, Thimphu, where they were stored at − 80 °C while awaiting testing.

#### *H. pylori* stool antigen test

Two rapid immunochromatography test (ICT) kits were used to detect the *H. pylori* antigen in the children’s stool sample. The first was the Quick Navi^™^ ICT kit (O-ICT), Lot No D9-212-4/R0, manufactured by Denka Co., Ltd. (Niigata Prefecture, Japan) and distributed by Otsuka Pharmaceutical Co., Ltd. (Tokyo, Japan). This commercial ICT kit has been available in the Japanese market since April 2021.

The second test kit referred to as A-ICT, is a novel immunochromatography assay developed by ADTEC Inc. (Usa City, Oita Prefecture, Japan) in collaboration with Oita University, Japan. This kit was custom-designed for use in Bhutan. Initially manufactured and assembled in Japan, the A-ICT kits were shipped to Bhutan for validation testing, with plans to gradually transfer production capacity to the country. Locally manufactured test kits are expected to provide significant advantages, including improved cost-effectiveness (reducing production and transportation expenses) and compliance with Bhutanese import regulations. Furthermore, the A-ICT’s simplified design requires minimal technical training and infrastructure, making it especially suitable for resource-limited settings. Unlike the individually packaged O-ICT kits, the A-ICT employs a multi-cassette sheet format that reduces packaging waste and facilitates batch processing. This design is particularly advantageous for primary health centers where supplies and personnel are limited.

**Fig. 1:**
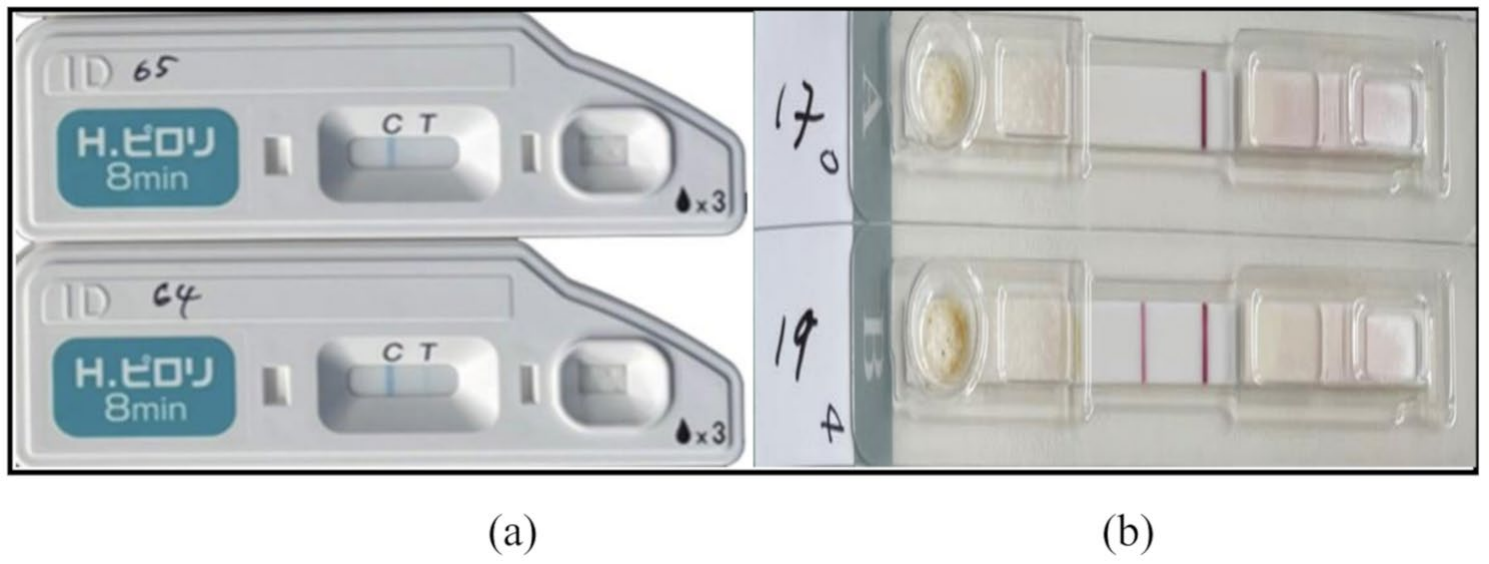
(**a**): Quick Navi^™^ sample 64 (+) and sample 65 (−) test. Picture dimensions: H = 3.88”, W = 5.94”; (**b**): ADTEC Inc., Japan sample 19 (+) and sample 17 (−) test. Picture dimensions: H = 4.66”, W = 3.77” The test pad works on the principle of lateral flow assay. The analyte moves along the pad, binding to gold-conjugated anti-*H. pylori* antibodies. If *H. pylori* antigens are present at the test line, a colored line appears. Unbound antibodies automatically bind to the control line, confirming that the test functions correctly. Results are read after 8 min (for test a) and 15 min (for test b). The appearance of both lines indicates a positive test result, whereas the appearance of only the control line indicates negative results. The appearance of only the test line indicates a faulty reading that necessitates a re-test

The O-ICT was conducted in accordance with the manufacturer’s instructions. Briefly, the samples were thawed at room temperature and diluted using the dilutant provided in the test kit. After adequate mixing, two drops were discarded and three were applied to the sample area on the rapid test kit. Consistent with the manufacturer’s instructions, the result was confirmed visually after 8 min (Fig. 1a).

The A-ICT kit for the SAT consists of a sample tube and a sheet containing ten ICT cassettes. The sample tube contains a 10 mL buffer solution and has a cotton-tip applicator attached to the screw cap of the tube on one end and a smaller exhaust tip closed by a smaller screw cap on the other. First, stool samples were thawed at room temperature. The cotton-tip applicator was dipped into the feces and allowed to be adequately coated. Next, the applicator was transferred into the buffer solution tube, which was closed tightly. Then, the sample was mixed thoroughly by manually shaking the tube (shaking away from the face to avoid accidents). Once mixed, the exhaust port was opened, and three drops of the mixture were applied to the sample area on the ICT. The test pad operates on the principle of lateral flow assay. Results were visually read after 15 min (Fig. 1b).

#### Statistical analysis

This study used SPSS version 25.0 (IBM Corp., Armonk, NY, USA) for data entry and cleaning and STATA version 14.2 (Stata Corp, TX, USA) for data analysis. Descriptive statistics such as percentage, mean, and standard deviation were used to describe the data. McNemar’s test was used to calculate sensitivity, specificity, positive predictive value, negative predictive value, and accuracy with a 95% confidence interval (CI). Agreement between the diagnostic tests (O-ICT and A-ICT) was evaluated using Cohen’s kappa coefficient in R, version 4.4.1. We conducted a logistic regression analysis to assess potential risk factors for *H. pylori* infection in children and presented the results as odds ratios (ORs) with their respective 95% CIs. The children’s nutritional status was analyzed using the World Health Organization software applications Anthro 3.2 and AnthroPlus 1.0.4. Statistical significance was assessed using a two-sided *p*-value threshold of less than 0.05.

Initially, the sample size was calculated based on an expected *H. pylori* infection prevalence of 66%, derived from a previous serological study conducted among Bhutanese children. However, the observed prevalence in the current study was considerably lower (19.54%). To evaluate whether the sample size remained adequate, a post hoc power analysis was performed using R (version 4.4.1), comparing the observed prevalence of 19.5% with the originally assumed rate of 66%. The effect size (Cohen’s *h*) was calculated to be 0.981 [22], yielding a statistical power of 1.0 at a significance level of 0.05. These results confirm that the sample size was sufficient to detect a statistically significant difference, despite the lower-than-expected prevalence.

## Results

### Comparison between the two ICT kits for the detection of *H. pylori* infection

**Fig. 2:**
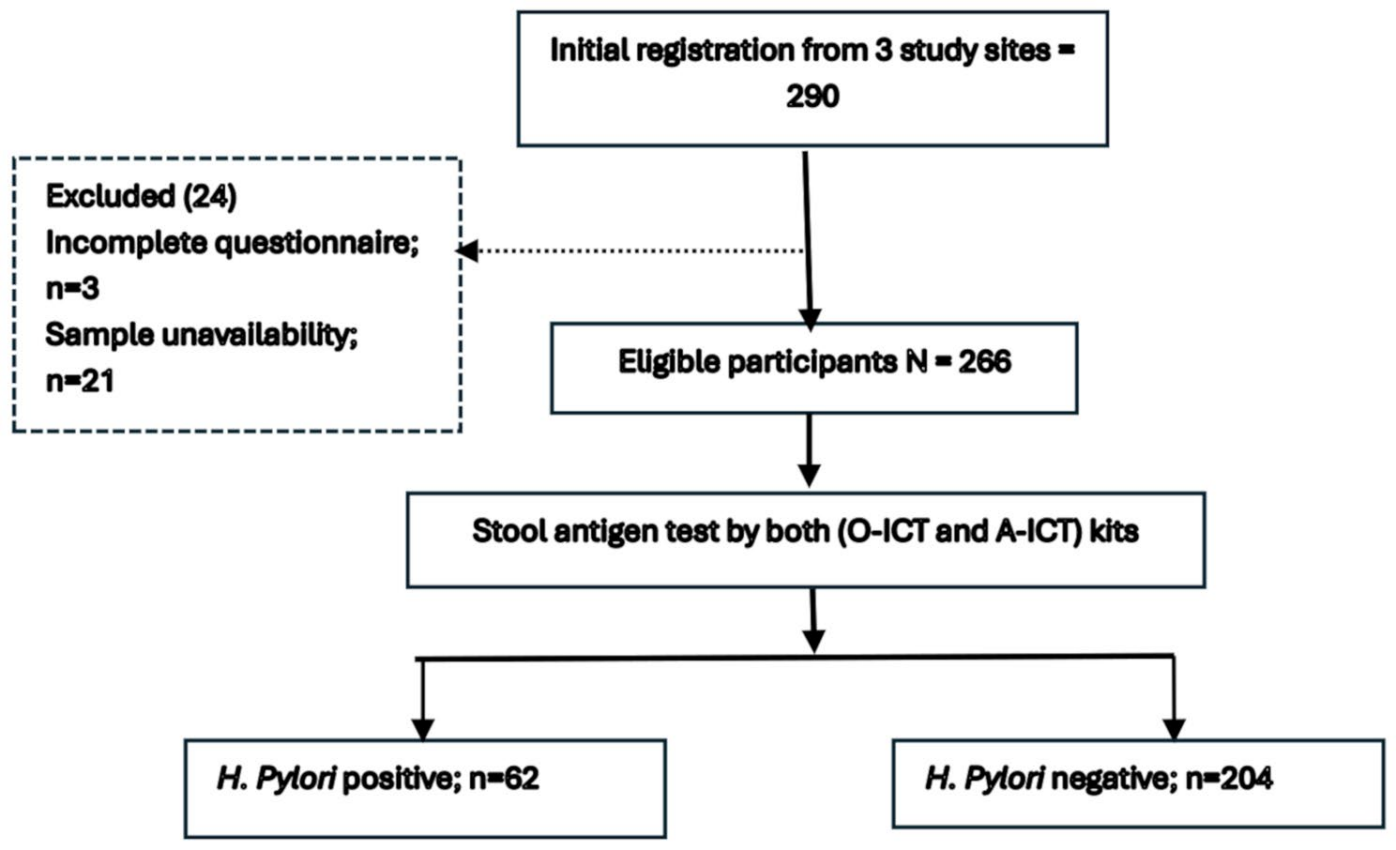
Flowchart of participant recruitment

Of the 290 registered participants, 24 were excluded, and 266 participants were finally included in the study (Fig. 2). The in-house A-ICT demonstrated high concordance with the commercial O-ICT kit (Kappa 0.84), and the overall agreement between the two test kits was 95.4% (Tables 1 and 2). The A-ICT exhibited excellent sensitivity (0.96; 95% CI: 0.910–1.00) and specificity (0.95; 95% CI: 0.93–0.98). The accuracy of the test was 0.95 (95% CI: 0.93–0.98), indicating a high level of reliability. Additionally, the positive predictive value was 0.83 and the negative predictive value was 0.99.

**Table 1:** Confusion matrix of the two diagnostic immunochromatography (ICT) kits for *H. pylori*

Test kits	O-ICT (+)	O-ICT (−)	Total
A-ICT (+)	50	10	60
A-ICT (−)	2	204	206
Total	52	214	266

**Table 2:** Sensitivity and specificity of the A-ICT kit

Parameters	Score	95% CI
Kappa	0.84	0.78–0.89
% agreement	95.4	0.75–0.93
Sensitivity	0.96	0.91–1.00
Specificity	0.95	0.93–0.98
Accuracy	0.95	0.93–0.98
PPV	0.83	0.74–0.93
NPV	0.99	0.98–1.00

PPV = Positive Predictive Value NPV = Negative Predictive Value

### Participant sociodemographic characteristics

Of the 266 participants, most were from Lungtenphu Hospital (69.5%), followed by Gidakom Hospital (18%) and Kuzugchen PHC (12.4%). One hundred and thirty-six participants (51.1%) were of the female sex. Most of the participants were between 48 and 60 months (24.1%), followed by those aged 24–36 months (20.7%) and 1–12 months (19.5%). The proportions of 36–48-month-olds (15.4%) and 12–24-month-olds (14.7%) were similar, and 60–64-month-olds (5.6%) were the least represented.

### Prevalence of *H. pylori* infection

In this study, *H. pylori* infection was defined as positive when it was tested positive on the validated commercial test kit: O-ICT. According to this criterion, the prevalence of *H. pylori* infection with the rapid stool antigen tests was (58/266) 19.54% (95% CI:14.95–24.83) among children aged 0–64 months in Thimphu, Bhutan. Although not statistically significant, the prevalence was slightly higher in boys (21.54%) than in girls (17.65%; Table 3).

**Table 3:** Binary and multivariable analysis of factors related to the prevalence of *H. pylori* in children

Variables	Categories	*H. pylori* stool antigen test result by ICT		COR (95% CI)	*P*-value	AOR (95% CI)	*P-*value
		Positive	Negative				
		*n* = 52 (%)	*n* = 214 (%)				
Age (months)	0–12	3 (3.85)	50 (96.15)	Ref		Ref	
	12–24	6 (15.38)	33 (84.62)	4.55 (0.86–23.95)	0.074	4.82 (0.83–27.99)	0.079
	24–36	16 (29.09)	39 (70.91)	10.25 (2.22–47.29)	0.003^**^	7.48 (1.38–40.43)	0.019^**^
	36–48	8(19.31)	33 (80.49)	6.10(1.21–30.33)	0.028^*^	3.50 (0.52–23.86)	0.2
	48–60	16 (25.00)	48 (75.00)	8.33(1.81–38.19)	0.006^**^	4.05 (0.63–25.88)	0.139
	60–64	4 (26.27)	11 (73.33)	9.10 (1.47–356.02)	0.017^*^	4.05 (0.53–30.66)	0.175
Sex	Male	28 (21.54)	102 (78.46)	Ref			
	Female	24 (17.65)	112 (82.35)	0.78 (0.42–1.43)	0.424		
Health center	Lungtenphu Hospital	38(20.54)	147 (79.46)	Ref			
	Kuzugchen PHC	8 (24.24)	25 (75.76)	1.23 (0.52–2.96)	0.632		
	Gidakom Hospital	6 (12.50)	42 (87.50)	0.55 (0.21–1.39)	0.21		
Birth order	First-born	15 (16.67)	75 (83.33)	Ref			
	Not first-born	37 (21.20)	139 (78.98)	1.33 (0.68–2.58)	0.85		
Number of siblings	One or no siblings	19 (13.38)	123 (88.62)	Ref			
	≥ 2 siblings	33 (26.61)	91 (73.39)	2.37(1.25–4.59)	0.008^*^	2.74 (1.27–5.91)	0.010^*^
Family type	Nuclear	44 (21.89)	157 (78.11)	Ref			
	Combined	8 (12.31)	57 (87.69)	0.50 (0.22–1.12)	0.095		
Number of people in household	4 people	27 (20.0)	108 (80.00)	Ref			
	≥ 4 people	25 (19.08)	106 (80.92)	0.94 (0.51–1.73)	0.851		
Primary caregiver	Mother/father	50 (20.16)	198 (79.84)	Ref			
	Other	2 (11.11)	16 (88.89)	0.49 (0.11–2.22)	0.359		
How the child is fed	Eat by self	31 (28.44)	78 (71.56)	Ref			
	Fed by others	21 (13.38)	136 (86.62)	0.38(0.21–0.72)	0.003^**^	1.11 (0.50–2.43)	0.79
Method of feeding	Eat with/fed using fingers	18 (29.03)	44 (70.97)	Ref			
	Eat with / fed using a spoon	34 (16.67)	170 (83.33)	0.48 (0.25–0.94)	0.034^*^	0.41 (0.19–0.91)	0.029^*^
Chewing child’s food	Yes	6 (30.00)	14 (70.00)	Ref			
	No	46 (18.70)	200 (81.30)	1.8 (0.67–5.10)	0.227		
GI symptoms	None	28 (19.31)	117 (80.69)	Ref			
	Stomachache	5 (14.29)	30 (85.71)	0.69 (0.24–1.95)	0.429		
	Diarrhea	19 (22.09)	67 (77.91)	1.18 (0.61–2.29)	0.621		
Father’s education	None	16 (21.33)	59 (78.67)	Ref			
	Traditional education	4 (13.79)	25 (86.21)	0.59 (0.17–1.94)	0.385		
	Primary	15 (21.43)	55 (78.57)	1.00 (0.45–2.22)	0.989		
	High school	17 (18.48)	75 (81.52)	0.83 (0.39–1.79)	0.645		
Mother’s education	None	15 (18.75)	65 (81.25)	Ref			
	Traditional education	4 (16.00)	21 (84.00)	0.82 (0.24–2.76)	0.755		
	Primary	9 (24.32)	28 (75.68)	1.39 (0.54–3.55)	0.489		
	High school	24 (19.35)	100 (80.65)	1.04 (0.51–2.12)	0.915		
Father’s occupation^¥^	Self–employed	4 (5.88)	64 (94.12)	Ref			
	Farmers/wager	3 (30.00)	7 (70.00)	4.43 (0.90–21.74)	0.067	9.17 (1.35–62.19)	0.023
	Government job	34 (24.29)	106 (75.71)	4.13 (1.66–10.32)	0.002**	4.66 (1.40–15.43)	0.012^*^
	Private employee	11 (22.92)	37 (77.08)	3.84 (1.33–10.99)	0.012*	6.73 (1.71–26.49)	0.006^*^
Mother’s occupation^¥^	Self–employed	4 (12.90)	27 (87.10)	Ref			
	Homemaker	39 (20.00)	156 (80.00)	1.61 (0.58–4.41)	0.36		
	Government job	4 (30.77)	9 (69.23)	2.31 (0.50–10.54)	0.279		
	Private employee	5 (18.52)	22 (81.48)	1.81 (0.50–6.59)	0.362		
Use of toilet by child	Use toilet by self	37 (25.34)	109 (74.66)	Ref			
	Use diaper	15 (12.50)	105 (87.50)	0.42 (0.22–0.81)	0.010^*^	0.52 (0.18–1.46)	0.221
Toilet location	Inside residence	38 (18.91)	163 (81.09)	Ref			
	Outside residence	14 (21.54)	51 (78.46)	1.17 (0.59–2.34)	0.46		
Toilet type	Automatic	38 (22.89)	128 (77.11)	Ref			
	Pour flush	14 (14.00)	86 (86.00)	0.54 (0.28–1.07)	0.079		
Water supply	Inside residence	46 18.85)	198 (81.15)	Ref			
	Collect from outside	6 (27.27)	16 (72.73)	1.61 (0.59–4.35)	0.95		
Drink open-stream water	Often	7 (35.00)	13 (65.00)	Ref			
	Never	45 (18.29)	201 (81.71)	0.42 (0.16–1.10)	0.077		
Boil drinking water	All the time	48 (20.60)	185 (79.40)	Ref			
	Not all the time	4 (12.12)	29 (87.88)	0.53 (0.17–1.58)	0.257		
Hand- washing using soap	All the time	49 (19.44)	203 (80.56)	Ref			
	Not all the time	3 (21.43)	11 (78.57)	1.12 (0.30–4.20)	0.855		
Hand-washing after changing the child’s diaper	All the time	48 (20.08)	191 (79.92)	Ref			
	Not all the time	4 (14.81)	23 (85.19)	0.69 (0.22–2.09)	0.515		
SAT of parents	Positive	3 (17.65)	14 (82.35)	Ref			
	Unsure/not tested	49 (19.68)	200 (80.32)	0.87 (0.24–3.16)	0.838		
Meat (beef, pork, and chicken) intake	Almost daily	2 (66.67)	1 (33.33)	Ref			
	3–4 times/week	3 (15.79)	16 (84.21)	0.09(0.00–1.39)	0.086		
	< once/week	47 (19.26)	197 (80.74)	0.11 (0.01–1.34)	0.085		
Fruit intake	Almost daily	23 (17.69)	107 (82.31)	Ref			
	3–4 times/week	30 (31.58)	65 (68.42)	1.97 (1.00–3.85)	0.047^*^	1.14 (0.50–2.60)	0.742
	< once/week	9 (21.95)	32 (78.05)	1.64 (0.67–3.98)	0.272	1.03 (0.36–2.91)	0.953
Vegetable intake	Almost daily	10 (11.90)	74 (88.10)	Ref			
	3–4 times/week	36 (31.03)	80 (68.97)	4.01(1.67–9.63)	0.002^**^	2. 62 (0.87–7.88)	0.085
	< once/week	16 (24.24)	50 (75.76)	2.96 (1.11–7.86)	0.029	1.73 (0.53–5.60)	0.358
Salty snack intake	Almost daily	3 (20)	12 (80.00)	Ref			
	3–4 times/week	14 (29.17)	34 (70.83)	1.18 (0.28–4.98)	0.813		
	< once/week	45 (22.17)	158 (77.83)	0.92 (0.24–3.42)	0.903		
BMI	Normal	38 (19.68)	155 (80.31)	Ref			
	Wasting	2 (18.18)	9 (81.82)	0.90 (0.18–4.36)	0.903		
	Overweight	12 (19.35)	50 (80.65)	0.97 (0.47–2.01)	0.954		
ZHA	Normal	33 (16.42)	168 (83.58)	Ref			
	Stunted	19 (29.23)	46 (70.77)	2.10 (1.09–4.03)	0.196		
ZWH	Normal	31 (18.67)	135 (81.33)	Ref			
	Wasting	2 (15.38)	11 (84.62)	0.79 (0.16–3.75)	0.769		
	Overweight	19 (21.84)	68 (78.16)	1.21 (0.64–2.31)	0.549		

^*^p < 0.05, ^**^p < 0.005. ^¥^Parents’ occupation was categorized as [1] self–employed (such as owning a small business, weaving, or doing carpentry work) [2], farmers [3], government jobs (e.g., in the military and hospitals), or [4] private employee (working in a privately owned company). Most mothers were homemakers and not farmers; therefore, in the mother’s occupation category, “farmer” was replaced with “homemaker.” AOR = adjusted odd ratio BMI = body mass index, CI = confidence interval, COR = crude odd ratio GI = gastrointestinal, ICT = immunochromatography test, SAT = stool antigen test, ZHA = Z-score height-for-age, ZWH = Z-score weight-for-height

### Sociodemographic factors and H. pylori infection

First, binary logistic regression was performed to assess the potential risk factors associated with *H. pylori* infection. The significant factors at *p* < 0.05 were further subjected to multivariable analysis. The factors associated with *H. pylori* prevalence after adjustment for the confounder are presented below.

Among the sociodemographic variables, the progressing age of children, having two or more siblings, and the father’s occupation were identified as significant risk factors for infection. In the univariate analysis, all age groups—except for the 12–24 months group—were significantly associated with *H. pylori* infection when compared to the reference group (0–12 months). However, in the multivariable analysis, only the 24–36 months groups remained significantly associated with infection. Notably, children aged 24–36 months had more than seven times the odds of *H. pylori* infection compared to those aged 0–12 months (AOR = 7.48; 95% CI: 1.38–40.43; *p* = 0.019) (Table 3). Children with two or more siblings had double the risk factor for infection compared with children with one or no siblings (AOR = 2.7; 95%CI: 1.27–5.9) (Table 3).

**Fig. 3:**
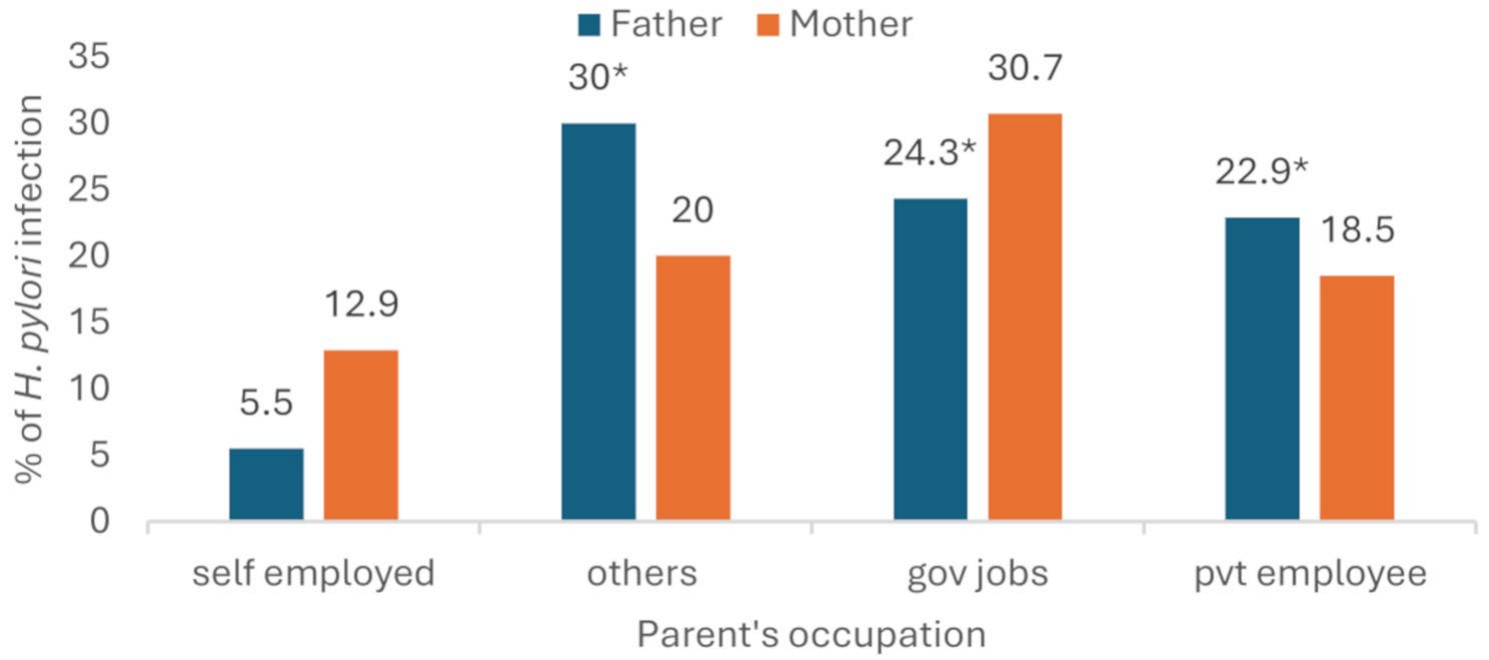
Parents’ occupation and *H. pylori* infection in children (^*^p < 0.05). Here “others” refers to farmers/wager as father’s and homemakers for mothers’ occupation respectively

The father’s occupation was identified as a statistically significant risk factor for *H. pylori* infection in children. Children whose fathers were farmers or wage earners had nine times the odds of *H. pylori* infection compared to those whose fathers were self-employed (AOR = 9.0; 95% CI: 1.35–62.19; *p* < 0.05). Similarly, children whose fathers were employed in government or private sectors had 4.6 (AOR = 4.6; 95% CI: 1.40–15.43; *p* < 0.05) and 6.7 times (AOR = 6.7; 95% CI: 1.71–26.49; *p* < 0.05) higher odds of infection, respectively, compared to those whose fathers were self-employed (Table 3). Although these associations were statistically significant (*p* < 0.05; Fisher’s exact test *p* = 0.006), the precision of these estimates may be limited due to substantial variability in the data and the relatively small number of observations within certain occupational categories (Table 3). The same tendency was observed with the mother’s occupation. However, children of the mothers consistently showed higher *H. pylori* infection than the children of fathers in each work group, and the difference between job groups was insignificant (Fig. 3). Other factors, including the type of primary caregiver, household size, gastrointestinal symptoms, and parents’ education, were not associated with infection.

### Environmental factors and *H. pylori* infection

Environmental factors such as sanitation and hygiene were assessed. Children still in diapers were less likely to test positive compared with those who used the toilet independently (COR = 0.4; 95% CI: 0.22–0.8); however, the difference was insignificant after adjustment (Table 3). Other factors such as location and type of toilet, water supply within or without the dwelling place, boiling water before consumption, washing hands with soap, or parents’ *H. pylori* status were not significantly associated with the prevalence of *H. pylori* infection in the children.

### Child feeding methods and *H. pylori* infection

Food-feeding methods were important in children who tested positive for *H. pylori* infection. The use of utensils such as spoons to feed children or the use of spoons by children significantly reduced the risk of *H. pylori* infection compared with the use of fingers to eat or feed (AOR = 0.41; 95% CI: 0.19–0.91; *p* < 0.05). Similarly, children fed by their mothers or other adult relatives had a lower risk than those who ate alone (COR = 0.38, 95% CI: 0.21–0.72; *p* < 0.05); however, the difference was insignificant after adjustment. Other factors, such as the pre-mastication of food before feeding the child, were not associated with the risk of infection (Table 3).

### Dietary factors and *H. pylori* infection

Dietary habits were assessed using a food frequency recall method. Univariate analysis revealed that not consuming fruits daily was significantly associated with a higher risk of *H. pylori* infection. Similarly, not eating vegetables on a daily basis was linked to increased odds of infection. Children whose parents reported providing fruits 3–4 times per week were more likely to be infected with *H. pylori* compared to those whose parents provided fruits daily (COR = 1.97; 95% CI: 1.00–3.85; *p* < 0.05). Likewise, the odds of *H. pylori* infection were higher among children whose parents reported providing vegetables 3 - 4 times per week (COR = 4.01; 95% CI: 1.67–9.63; *p* < 0.05) and less than once per week (COR = 2.96; 95% CI: 1.11–7.86; *p* < 0.05), compared to those who received vegetables daily (Table 3). However, multivariable analysis did not show statistical significance. Other factors, including the consumption of salty foods or meat, showed no association with infection. Logistic regression showed no association between children’s nutritional status and *H. pylori* prevalence (Table 3).

## Discussion

This cross-sectional study determined the prevalence of *H. pylori* infection among children under 64 months of age in Bhutan while evaluating the efficacy of the rapid in-house A-ICT kit. ICT kits are well established and recommended as first-line screening tools for detecting active *H. pylori* infection and confirming eradication [23–25]. In this study, the A-ICT kit demonstrated a sensitivity of 96.2% and a specificity of 95.3%, with a 95.4% agreement and a Kappa of 0.84 compared with the O-ICT kit, indicating its robustness for clinical use [26]. Long-term follow-up in randomized controlled trials consistently showed a significantly reduced incidence of GC among populations who have undergone *H. pylori* eradication therapy compared with those who have not received therapy [27, 28]. A cost analysis in Japan, a country at high risk for GC, suggested that a population-based *H. pylori* eradication strategy is a more optimal and cost-effective approach as a national GC prevention program than an endoscopic method for secondary prevention [29]. These findings indicate that early diagnosis and eradication of *H. pylori* can significantly reduce GC incidence and be more cost-efficient than secondary prevention strategies. The WHO recommends early eradication of *H. pylori* in children and young adults as a strategy to prevent gastric cancer, noting that it is more feasible and cost-effective than mass eradication efforts in adults, which are complicated by high costs and concerns regarding antimicrobial resistance [30]. Given its non-invasive nature and comparable ease of use to conventional ICT kits, the A-ICT shows strong potential for use in resource-limited settings like Bhutan. Its two main advantages are [1] an innovative multi-use sheet design with minimal packaging, which reduces waste and improves logistics; and [2] the potential for local production, reducing reliance on imports. Although a formal cost-effectiveness analysis has not yet been conducted, these features—together with the kit’s high diagnostic accuracy—position the A-ICT as a sustainable tool for scalable *H. pylori* screening. It may also support long-term national strategies for gastric cancer prevention in Bhutan. Moreover, unlike imported O-ICT kits, the A-ICT can be manufactured locally, which enhances long-term sustainability, reduces dependency on international supply chains, and ensures accessibility even in remote regions.

The study demonstrated a substantial decline in the prevalence of *H. pylori* infection in children—from 66% in previous reports to 19.54% in the present study [15]. This reduction may be attributed to an overall improvement in living standards, such as better sanitation, hygiene, housing conditions, nutritional status [31], and the recently implemented eradication program under the national cancer flagship program [17]. However, it is important to note that the earlier estimate was based on serological testing and included participants aged 4 to 19 years. In contrast, the current study assessed active infection using a stool antigen test in children under 64 months of age. Although the observed prevalence was considerably lower than initially anticipated, a post hoc power analysis confirmed that the final sample size (*n* = 266) was sufficient to detect large effect sizes. These findings support the adequacy of the sample size for identifying meaningful associations, even with the lower-than-expected infection rate. The youngest age at which *H. pylori* was detected was 9 months, and infection was lowest among 0–12-month-olds and highest among 24-36-month-olds (Table 3). Meta-analysis of global prevalence revealed that the rate of *H. pylori* infection ranged from 24% (95% CI: 19.5–29.2) in children under 6 years to over 43% (95% CI: 37.1–50.2) in those aged 13–18 years [14]. In our study, although univariate analysis demonstrated increasing age as a significant risk factor for *H. pylori* infection in all age groups, multivariable analysis showed significance in the 24–36-month (COR = 10.25%; 95% CI: 2.22–47.29). However, the large variability in the data set suggests that further studies with larger sample sizes are needed to confirm increasing age as a risk factor for *H. pylori* infection among Bhutanese children. Nonetheless, the findings reflect that exposure to infectious sources increases as children grow. Having two or more siblings was a risk factor for *H. pylori* infection, consistent with the finding of previous reports in which having three or more children per household was a significant risk factor for infection (OR = 1.4; 95% CI:1.0–1.9) [15]. Infected family members are a potential risk factor for infection in children [14]. The Maastricht VI consensus [32] recommends eradication in young adults, particularly women, to reduce the risk of intra-familial *H. pylori* transmission to children. The consensus suggests that eradicating *H. pylori* at a young age to prevent GC is particularly beneficial given its impact decreases with age. Integrating *H. pylori* screening using rapid ICT kits in young adults can be an effective and affordable primary GC control strategy in Bhutan.

Feeding children using cutlery was more protective against *H. pylori* infection than feeding using fingers. Similar contextual findings were reported in China, where sharing cutlery between a feeding person and young children rather than using separate cutlery was associated with the risk of *H. pylori* infection in children (OR = 1.43, 95% CI:1.01–2.01) [7]. These findings establish the plausibility of ingesting the organism through the gastro-oral or oral-oral route and most probably indicate inadequate hygiene practices [33].

In contrast to the previous finding of an inverse relationship between the mother’s education and infection prevalence [15], this study identified the father’s employment status as a risk factor. Similar risk factors have been reported in other parts of the world [34]. The findings of this study may be attributed to the specific occupation of the fathers given that most of the children in this study were from military families (with the fathers working in the military), followed by children from the Gidakom Hospital area. In this industrial region, fathers either worked in the limestone and sand factory that belongs to the private sector or in the hospital as sanitary workers or night guards. A literature review [35] on *H. pylori* infection among military personnel reported that the highest prevalence of infection (50.2%; 95% CI: 31–33) in Asia is attributable to familial aggregation and poor living environments. Similarly, Kheyre et al. [36] observed that health workers, sewage workers, and miners were at higher risk of acquiring *H. pylori* infection than those in other professions. This study examined a broad range of parental occupations and did not define specific occupations such as military personnel, sand factory worker, or hospital employee. Despite significant results, the high variability in the data limited accuracy. Future studies with large sample sizes are needed to better estimate prevalence and confirm whether the father’s occupation is a risk factor for *H. pylori* infection in children.

Although univariate analysis showed a statistically significant association between low intake of fruits and vegetables and the risk of infection, this association became insignificant after adjustment for confounders. However, studies in China [37] and Iran [8] have demonstrated a potential link between vegetable and fruit consumption and reduced *H. pylori* infection risk. Fruits and vegetables significantly contribute to dietary fiber and boost short-chain-fatty acid-producing bacteria, enhancing intestinal barrier function, maintaining homeostasis, and preventing bacterial adhesion and invasion. Moreover, flavonoids and vitamins, especially vitamin C from fruits and vegetables, act as anti-inflammatory and antioxidant agents that augment epithelial integrity [38, 39]. These mechanisms suggest that fruits and vegetables protect against *H. pylori* infection by enhancing immunity at multiple levels. Our findings did not confirm this association; further studies are warranted to investigate this potential protective role. Moreover, this study did not show a statistical association between nutritional status and the prevalence of *H. pylori* infection. Similar results were reported in Bangladesh among 12–18-month-old infants in whom *H. pylori* infection was not associated with childhood growth indicators [40].

### Limitations

This study is not without limitations. First, the study was conducted in the peri-urban and urban areas of the capital city of Thimphu and may not represent the entire country. Second, the sample size may have limited the ability to precisely detect some associations, as suggested by the variability observed in specific analyses. A larger sample size could have improved the precision of estimates and confirmed the associations between variables. Finally, the environmental conditions in the capital city differ from those in rural villages, limiting the generalizability of risk factors to the entire population.

## Conclusions

This study demonstrated that the A-ICT has high sensitivity (96.2%) and specificity (95.3%), making it an effective diagnostic tool. The validation of the test kits represents a significant step toward enhancing the diagnosis and eradication of active *H. pylori* infection in Bhutan. Despite a significant reduction in *H. pylori* prevalence, the increasing infection rate with age, especially among the 24–36-month age group, remains a potential risk factor for infection. Additionally, the study underscored the importance of hygiene practices—particularly those related to child feeding—in protecting children from the risk of *H. pylori* infection. These findings emphasize the need to enhance sanitation and hygiene at all levels, including that of individuals, households, and workplaces. Population surveillance strategies, especially those targeting young adult and pediatric populations, are essential for eradicating *H. pylori* infection before it causes irreversible damage to the young population. Introducing the A-ICT to the Gastric Control Program can be an effective first-line screening tool because the A-ICT is non-invasive, easy to use, and reliable.

## Electronic supplementary material

Below is the link to the electronic supplementary material.

Supplementary Material 1

## Data Availability

No datasets were generated or analysed during the current study.

## Abbreviations

AOR: Adjusted odd ration
BMI: Body mass index
COR: Crude odd ratio
CI: Confidence interval
GC: Gastric Cancer
GI: Gastrointestinal
ICT: immunochromatography test
JICA: Japan International Cooperation Agency
PHC: Primary Health Center
SAT: stool antigen test
ZHA: Z-score height-for-age
ZWH: Z-score weight-for-height
