# Supplementary material for: Prevalence of and risk factors for Helicobacter pylori infection in children under 64 months in Thimphu, Bhutan, and introducing the new in-house immunochromatography test kit: a cross-sectional study
Source: Gut Pathog. 2025 Jun 4;17:39. doi: 10.1186/s13099-025-00715-2 (PMC12139328; doi:10.1186/s13099-025-00715-2)
Supplement: Supplementary file 1 — Supplementary Material 1 [file 13099_2025_715_MOESM1_ESM.docx]

**Questionnaire for a child six years and below and their primary caregivers for a study titled “Prevalence and risk factor of *Helicobacter pylori* infection among asymptomatic children in Thimphu Dzongkhag Bhutan”**

Date ___/____/____2023 Time:_____AM / PM

ID No: ___________________________ contact number ___________________

**Section 1: General information of the child**

**Note: (**ideally the primary caregiver will answer the question**)**

| 1. Date of birth: Date ­­­_______month ______year_______ (from the MCH record) |
| --- |
| 1. Sex: Male  female |
| 1. Length (for less than 2 years) / height ____________cm |
| 1. Weight: _________kg |
| 1. Number of siblings: |
| 1. Birth order: |
| 1. Number of people living in the same house with the child:   (relationship with the child: father/mother/grandma/grandpa/uncle/aunt/sibling/cousin) – tick all that is applicable |
| 1. Who is the primary care giver of the child? (Tick all that applies)   [1 ] Mother  [ 2 ] Father  [ 3 ] Grandmother  [ 4 ] grandfather  [ 5 ] Baby sitter  [ 6 ] Other relatives |
| 1. Mostly, who is responsible for feeding food to the child?   [ 1] feed by himself/herself  [ 2] Mother  [ 3] Father  [ 4] Grandmother  [ 5] grandfather  [ 6] Baby sitter  [7 ] Other relatives |
| 1. How is the child fed?   [1] child eat by himself/herself using fingers  [ 2 ] child eat by himself/herself using spoon  [ 3 ] someone feeds the child using their fingers  [4 ] someone feeds the child using a spoon |
| 1. Does the mother or someone chew food (meat / zaw etc) and feed this food to the child?   [ 1] Yes  [ 0 ] No |
| 1. Within the last one months did you experience (did the child ever complained of) any of the following?   [1] Stomach ache  [2] Nausea (feeling of vomiting, along with drooling)  [3] Gastroesophageal regurgitation (food flowing back to your throat when you burp)  [4] Epigastric pain (pain in the upper region of your stomach)  [5] Bloating (feeling of fullness and discomfort even without eating)  [6] Diarrhoea  [7] constipation |

**Section 2: Types of food**

|  |  | Almost daily | 3-4times per week | Once or twice per week | Less than once or twice a month | Almost never |
| --- | --- | --- | --- | --- | --- | --- |
| 1. **How often do you feed the following foods to your child?** | | | | | | |
| 1. Fresh meat (beef/pork/chicken) | |  |  |  |  |  |
| 1. Koka /maggi/wai wai | |  |  |  |  |  |
| 1. Other instant noodles and chips | |  |  |  |  |  |
| 1. All king of vegetables: (green leafy, broccoli, tomatoes, asparagus, cabbages, green beans, peas, Pumpkin, carrots) | |  |  |  |  |  |
| 1. All kinds of fruits: (Guava, apples, oranges, grapes, pineapple, papaya, peaches) | |  |  |  |  |  |

**Section 3: Caregiver’s and household information**: Place a tick [✓] in the box you feel most appropriate).

| 1. Father’s education level:   [ 1 ] Illiterate  [ 2 ] Non-formal  [ 3 ] Primary level  [ 4 ] High school  [ 5 ] College/university  [ 6 ] Others (specify): |
| --- |
| 1. Father’s occupation:   [ 1 ] unemployed  [ 2 ] daily wage earner  [ 3 ] Farmer  [ 4 ] Civil service (govt. employee)  [ 5 ] Corporate employee  [ 6 ] Private employee  [ 7 ] Other (specify)s: |
| 1. Mother’s education level:   [ 1 ] Illiterate  [ 2 ] Non-formal  [ 3 ] Primary level  [ 4 ] High school  [ 5 ] College/university  [ 6 ] Others (specify): |
| 1. Mother’s occupation:   [ 1 ] unemployed /homemaker  [ 2 ] daily wage earner  [ 3 ] Farmer  [ 4 ] Civil service (govt. employee)  [ 5 ] Corporate employee  [ 6 ] Private employee  [ 7 ] Other (specify |
| 1. Where is the toilet located in your house?   [ 1 ] inside the house  [ 2 ] outside the house  [ 3 ] no toilet |
| 1. Type of toilet at home   [ 1 ] Pit  [ 2 ] Flush  [3 ] pour flush  [ 4 ] Shared toilet  [ 5 ] no toilet |
| 1. Drinking water supply   [ 1 ] pipe water inside the house  [ 2 ] pipe water outside the house  [ 3 ] pipe water share within community  [ 4 ] sources other than pipe water |
| 1. Where does your child go for toilet?   [ 1 ] in the adult toilet  [ 2 ] outside in the drain  [ 3 ] in the diaper  [ 4 ] others _______________________ |

**Section 4:** Individual behaviour for Water, sanitation and Hygiene (WASH)

Note: (the primary caregiver will answer the question)

| **Item No.** | **Behavioral WASH Items** | Always | Most of the time | Some  times | Rarely | Never |
| --- | --- | --- | --- | --- | --- | --- |
|  | **Water** | | | | | |
|  | How often do you drink tap water? |  |  |  |  |  |
|  | How often do you get drinking water directly from other sources such as river, stream, spring? |  |  |  |  |  |
|  | How often do you drink boiled water? |  |  |  |  |  |
|  | **Hygiene** | | | | | |
|  | How often do you wash hands before meals? |  |  |  |  |  |
|  | How often do you wash hands with soap? |  |  |  |  |  |
|  | How often do you wash hands after using the toilet? |  |  |  |  |  |
|  | how often do you wash hands before feeding your child? |  |  |  |  |  |
|  | How often do you wash hands after changing your child’s diapers |  |  |  |  |  |
|  |  |  |  |  |  |  |

**Section 5: For the primary care giver *H. pylori* status**

1. Relationship to the participant (child).
2. [ 1 ] Mother
3. [ 2 ] Father
4. [ 3 ] Others: specify (………………………………)
5. Did you ever give a stool for *H. pylori* test?
6. [ 1 ] Yes
7. [ 0 ] No

End of interview
